# SesameFG: an integrated database for the functional genomics of sesame

**DOI:** 10.1038/s41598-017-02586-3

**Published:** 2017-05-24

**Authors:** Xin Wei, Hao Gong, Jingyin Yu, Pan Liu, Linhai Wang, Yanxin Zhang, Xiurong Zhang

**Affiliations:** 1 Key Laboratory of Biology and Genetic Improvement of Oil Crops, Ministry of Agriculture of People’s Republic of China, Oilcrops Research Institute, Chinese Academy of Agricultural Sciences, Wuhan, 430062 China; 2 National Center for Gene Research, Collaborative Innovation Center for Genetics and Development, Institute of Plant Physiology and Ecology, Shanghai Institutes for Biological Sciences, Chinese Academy of Sciences, Shanghai, 200233 China

## Abstract

Sesame (*Sesamum indicum* L.) has high oil content, a small diploid genome and a short growth period, making it an attractive species for genetic studies on oilseed crops. With the advancement of next-generation sequencing technology, genomics and functional genomics research of sesame has developed quickly in the last few years, and large amounts of data have been generated. However, these results are distributed in many different publications, and there is a lack of integration. To promote functional genomics research of sesame, we collected genetic information combined with comprehensive phenotypic information and integrated them in the web-based database named SesameFG. The current version of SesameFG contains phenotypic information on agronomic traits of 705 sesame accessions, *de novo* assembled genomes of three sesame varieties, massive numbers of identified SNPs, gene expression profiles of five tissues, gene families, candidate genes for the important agronomic traits and genomic-SSR markers. All phenotypic and genotypic information in SesameFG is available for online queries and can be downloaded freely. SesameFG provides useful search functions and data mining tools, including Genome Browser and local BLAST services. SesameFG is freely accessible at http://ncgr.ac.cn/SesameFG/. SesameFG provides valuable resources and tools for functional genomics research and the molecular breeding of sesame.

## Introduction

Sesame (*Sesamum indicum* L.) is a major oilseed crop and is widely grown around the world, with concentrations in Asia and Africa^1^. With excellent characteristics, such as high oil content (~55%), strong drought resistance, a short growing season (~90 d), a large propagation coefficient (3,000–10,000 seeds per plant), and a small diploid genome (~350 Mb), sesame is regarded as an attractive model for genetic research of oilseed crops^2^. In addition, more than 35,000 accessions of sesame have been collected worldwide^3^, and massive genome variations have been identified in the sesame population^4, 5^. The rich levels of phenotypic and genotypic diversity of sesame are also valuable for functional genomics research.

With the development of next-generation sequencing (NGS) technology, genetic research on sesame has developed quickly in the last few years. Genome sequencing of both the variety and landrace sesame have been published^5, 6^, large amounts of polymorphism molecular markers have been developed^7–10^, several genetic linkage maps have been constructed, and many quantitative trait loci (QTL) have been identified^11–15^. Additionally, some gene families were investigated^16, 17^, gene expression profiles in different tissues were detected by transcriptome sequencing^6, 8, 18, 19^, and more than 5.4 million single nucleotide polymorphisms (SNPs) were identified from large-scale genome re-sequencing^2^. Candidate genes that are related to oil production and quality were explored in genome-wide association studies (GWAS), providing precise clues for uncovering the genetic mechanism of important sesame agronomic traits. These achievements in sesame genetic research provide a valuable basis for functional genomics research. However, these results are distributed over different publications and lack integration. It is difficult for researchers to utilize the previous results and data for future sesame research.

Three databases that are related to sesame genomics have been constructed in the last years, including Sinbase (http://ocri-genomics.org/Sinbase/)^20^, the Sesame Genome Project (http://www.sesamegenome.org/)^21^, and SesameHapMap (http://ncgr.ac.cn/SesameHapMap/)^2, 5^. The genome sequences of several sesame varieties and SNPs in the population are provided in these databases. The genomics-related databases laid the foundation for the construction of a functional genomics database. By combining the genomics data with newly released functional genomics data and integrated phenotype information, a comprehensive sesame functional genomics database can be constructed. Comprehensive and integrated databases for functional genomics research have been constructed in several other crops, such as rice, tomato, and foxtail millet^22–24^. These databases have been widely used in functional genomics research and have greatly promoted the basic research and molecular breeding of these crops^25–27^. Therefore, an integrated database will likely play an important role in sesame functional genomics research.

Germplasm collections contain superior alleles that can be uncovered by functional genomics research and used in crop breeding^28^. Generally, crop improvement relies on the utilization of superior alleles contained in the germplasm^29^. Collection of various germplasms and identification of admirable germplasms is the basis for crop functional genomics research and molecular breeding. To identify valuable germplasm with superior alleles, the precise phenotypic information of the germplasm is required. Many germplasm resources have been collected in several sesame germplasm banks^30–32^, and the important agronomic traits of these germplasms have been observed. However, little phenotypic information of these germplasms is available online. As far as we know, there is no database that contains detailed phenotype information of various sesame germplasms. Consequently, a database which includes comprehensive and detailed phenotype information of sesame germplasms is desired for researchers.

We have established the SesameFG (Sesame Functional Genomics Database, http://ncgr.ac.cn/SesameFG/) to provide comprehensive genetic information, phenotypic information and bioinformatics analysis for sesame functional genomics research. The published data, which were useful for gene identification and gene functional research in sesame, were collected and analyzed in our database, including materials information, genome sequences, genome variations, gene families, gene expression, candidate genes and simple sequence repeat (SSR) markers. The detailed phenotype of sesame core collections that precisely investigated by our group were also submitted and integrated into the database. In addition, the gene functional analysis tools, Genome Browser and BLAST, are available in the database. The goal of the database is to build a user-friendly and widely used repository that covers comprehensive functional genomics-related resources and that will be updated with newly released data regarding sesame functional genomics research in the future. In this study, we introduce the current version of SesameFG.

## Methods

### Data collection

**Figure 1:**
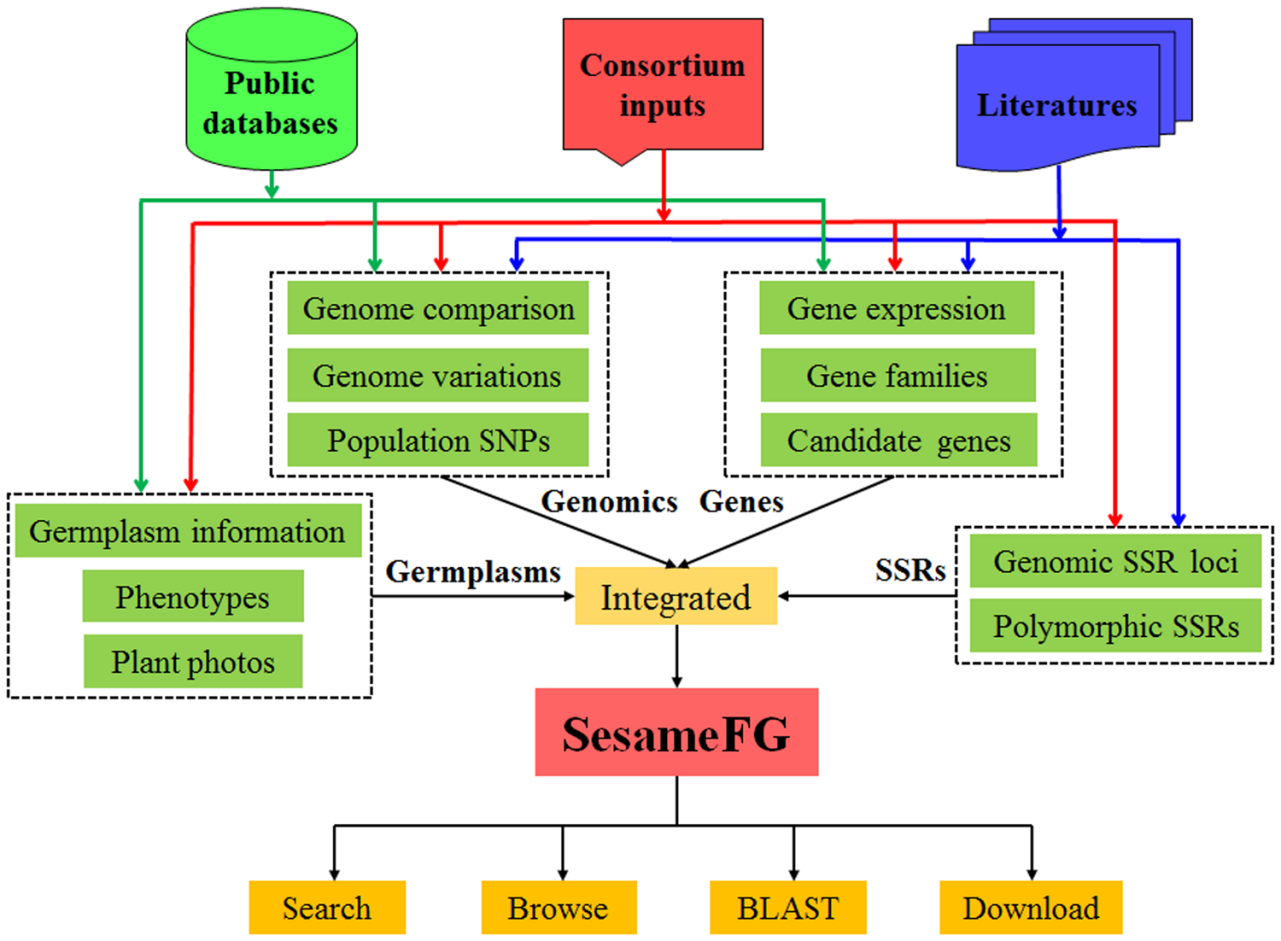
Flowchart of the construction of SesameFG. SesameFG is a comprehensive database for browsing and retrieving resources about sesame functional genomics and related data. SesameFG collected information mainly from three sources: i) genetic resources from public databases; ii) detailed curation of functional genomics research from the literature; iii) public contributions from the sesame functional research consortium and unpublished data from our group. All of the information stored in the database can be browsed in a user-friendly manner and downloaded through any web browser.

SesameFG was constructed using large-scale genetic and phenotypic sesame resources that came from public databases, literature and sesame functional genomics consortium inputs (Fig. 1). The genetic and phenotypic information collected and integrated in SesameFG mainly includes germplasm information, phenotypes, plant photos, genome sequences and variations, population SNPs, gene families, gene expression profiles, candidate gene of agronomic traits, SSR loci and polymorphic SSR markers (Table 1).

**Table 1:** Summary of the data sources.

Category	Description	Detail	Source	References
Genome	Zhongzhi13	274 Mb	Sinbase	6, 27
	Mishuozhima	254 Mb	SesameHapMap	5
	Baizhima	267 Mb	SesameHapMap	5
Genome variation	SNPs	1,332,025 SNPs	Publication	5
	Indels	506,245 Indels	Publication	5
	Transposons	525,537 transposons	Publication	5
	Population SNPs	5,407,981 SNPs	SesameHapMap	2
Germplasm information	Core collections	705 accessions	Publication	2
Phenotype	Agronomic traits	56 traits	Zhang’s group	
Gene expression	Difference in tissues	27,148 genes in 5 tissues	Zhang’s group and NCBI (SRA122023)	6
	Root under waterlogging stress	27,148 genes in 5 stress points	NCBI (SRX1406650)	18
	Stem tip and leaf of determinate growth plant	27,148 genes in 2 developmental stages	Zhang’s group	
	Developing seeds	27,148 genes in 5 developmental stages	Zhang’s group	
Gene family	MADS-box, AP2 and Hsf	219 genes	Publications	16, 17, 37
	Transcription factor and common gene families	3,867 genes of 26 gene families	Zhang’s group	
Candidate gene	Oil quality and production related genes	47 genes	Publication	2
SSR	Genomic SSRs	104,836 SSRs	Zhang’s group and publication	9
	Polymorphism SSRs	218 SSRs	Publications	7, 8, 9

#### Germplasm information and phenotypes

Sesame core collection information was collected in SesameFG, using accession name, geographic origin, ecotype, sequencing coverage and group information. The sesame core collection consists of 705 accessions from 29 countries around the world^33^, representing the most genetic diversity of all of the germplasms conserved in the Chinese Sesame Genebank of the Oil Crops Research Institute. All of these materials had their genomes re-sequenced with an average of ~2.6-fold genome coverage, and the results were used in GWAS research^2^. Phenotype values of fifty-six agronomic traits of the 705 sesame accessions in four environments were collected into SesameFG. The fifty-six traits included yield-related, disease resistance, quality, growth cycle related, and morphological traits. The four phenotyping sites included Luohe in Henan province (114.02E, 33.56N), Wuhan in Hubei province (114.30E, 30.60N), Nanning in Guangxi province (108.33E, 22.84N) and Sanya in Hainan province (109.31E, 18.14N). Moreover, photos of the flowering stage of each accession were also included.

#### Genome sequences and variations

The genome sequence of “Zhongzhi13”, a widely grown sesame variety, was downloaded from Sinbase (http://ocri-genomics.org/Sinbase/) and used as a reference genome^6^. The other two assembled genome sequences of sesame landraces, “Mishuozhima” and “Baizhima”, were obtained from SesameHapMap (http://ncgr.ac.cn/SesameHapMap/). In addition, 5,407,981 sesame population SNPs were also downloaded from SesameHapMap. These population SNPs were identified from the re-sequencing of 705 sesame core collections^2^.

#### Gene families

Gene families in the sesame genome were identified using several analysis tools, such as Pfam, HMM, BLAST and SMART^34–36^. In addition, all published sesame gene families, including MADS-box, AP2 and Hsf genes, were collected into SesameFG^16, 17, 37^.

#### Gene expression

The gene expression profiles of different tissues in several sesame accessions were collected^6^. These profiles included expression information for the capsule, leaf, seeds and stem of the sesame variety “Zhongzhi13”, for the root of the waterlogging-tolerant variety “ZZM2541”, for the stem tip of the typical determinate growth sesame accession “ZZM3305” and for the developing seeds of the high oil content variety “Zhongfengzhi1”. All gene expression levels were indicated by the Reads Per Kilobases per Million reads (RPKM) values, which were calculated from the transcriptome sequencing data.

#### Candidate genes of agronomic traits

Candidate genes that are related to important agronomic traits were collected and provided in SesameFG, such as yield, lipid metabolism, beginning flowering date and disease resistance. These genes were discovered in genetic analysis and molecular experiments^2^.

#### SSR markers

We identified SSR loci in sesame genomes using the microsatellite identification software (MISA)^38^ and designed SSR markers for each locus using Primer3^39^. One to six nucleotide motifs were considered, and the minimum repeat unit was defined as ten reiterations for mononucleotides, six reiterations for dinucleotides, and five reiterations for other repeat units. In total, 104,836 SSR markers were developed from the sesame genome, and all were submitted into SesameFG. In addition, the polymorphic SSRs that were detected from previous experimental research were also collected^7–9^.

### Data preprocessing

The assembled contigs of “Mishuozhima” and “Baizhima” were aligned on the “Zhongzhi13” genome by MUMmer^40^ to identify the homologous genome regions. The genome variants in “Mishuozhima” and “Baizhima” were detected using the diffseq program in the EMBOSS package^41^. The SNPs in each gene were identified, and the SNP effect was annotated using the reference genome. The transposons in “Mishuozhima” and “Baizhima” were identified using RepeatMasker (http://repeatmasker.org) and annotated by aligning with the “Zhongzhi13” genome.

To use whole-genome SNPs conveniently, each SNP of the population SNPs was labeled with a unique identifier (ID, e.g., Sis0000000011). The SNPs present in each gene were identified using a self-customized Perl script. The SNP number and their allele frequency in landraces, varieties, south and north groups were summarized. Moreover, the SNPs between each two accessions of the core collection were identified. The linkage group location, position, and numbers and frequency in different groups of SNPs were also identified. Moreover, the SNPs between each two accessions, which were valuable for identification of the variations in the genomes of the different sesame germplasms, were also analyzed and provided in the database.

The *Arabidopsis* genome was downloaded from the TAIR database^42^. All genes in the sesame genome were analyzed against *Arabidopsis* genes using BLAST and were annotated using homologous *Arabidopsis* genes^34^. For the gene expression profiles, the gene expression of each accession was integrated based on the gene ID in the sesame genome. Furthermore, the SSR loci in the sesame genome around each gene was identified and provided in the database.

The gene structure, tRNA genes, microRNA genes, transposons and other genome components downloaded from Sinbase and the population SNPs downloaded from SesameHapMap were integrated into the Genome Browser^43^. Detailed information of the genome components and population SNPs, such as the ID, position, length and sequences, were linked into the Browser.

### Database implementation

The SesameFG database was developed using Perl/CGI, Python and JavaScript on a platform with the MySQL 5.0 database management system. The web interfaces were constructed using PHP (Version 5.6), a popular scripting language for dynamic webpages. JavaScript and jQuery were used to enhance the website interface and to improve the user experience. A navigation tool bar containing several links is also contained in each webpage. The database is powered by an Apache server running Ubuntu Linux 15.04.

## Results

### The database feature of SesameFG

**Figure 2:**
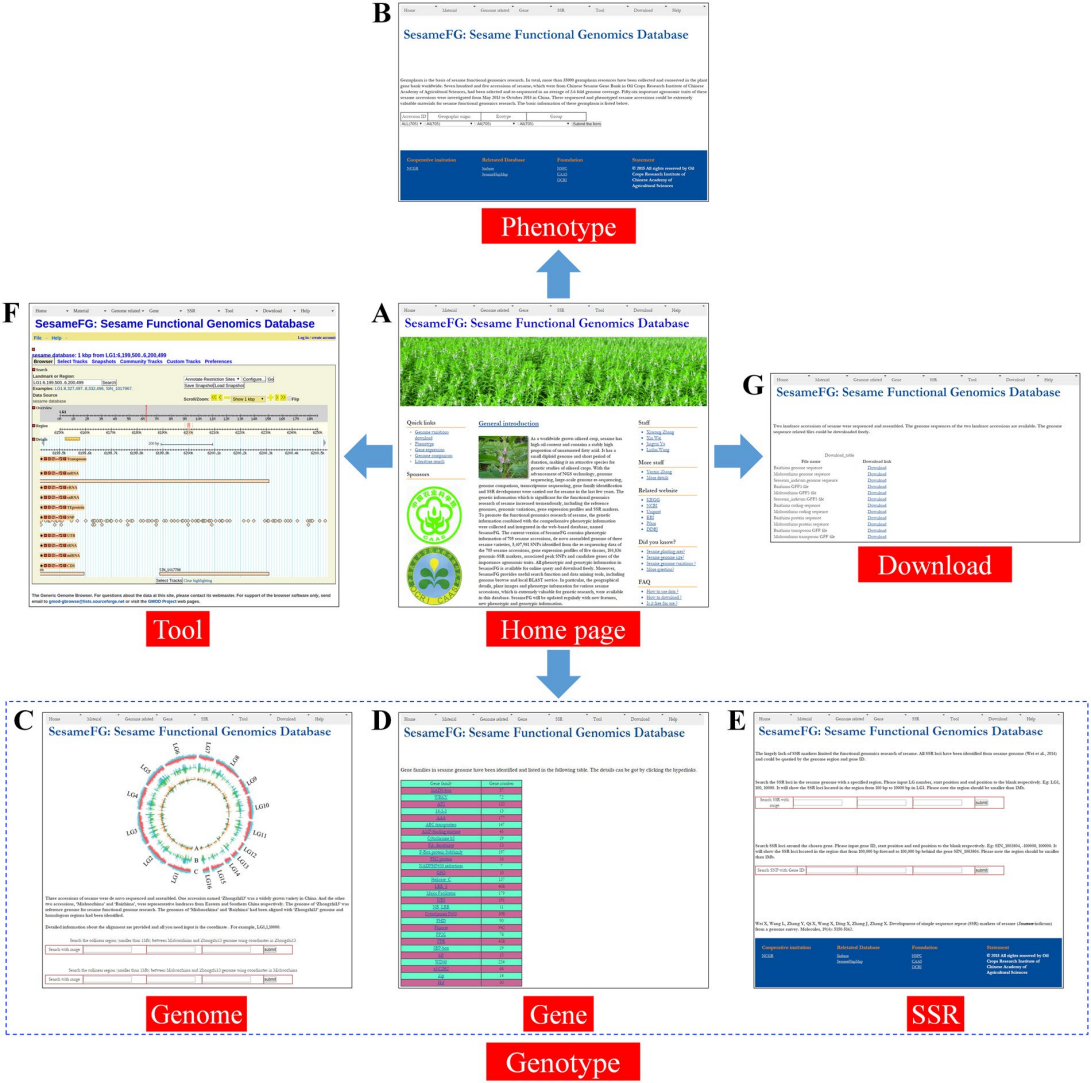
Main contents and interfaces of SesameFG. (**A**) The home page of SesameFG. (**B**) Basic information of the 705 sesame germplasm. (**C**) Collinear regions and genome variations in sesame genomes. (**D**) Gene families identified from the sesame genome. (**E**) SSR loci identified from the sesame genome. (**F**) The Genome Browser for locating gene structures, SNPs, and RNAs. (**G**) Genome variations of 705 sesame accessions for downloading.

To promote sesame functional genomics research, SesameFG has collected both the comprehensive genetic and phenotypic information of sesame and endeavors to provide all necessary resources and tools for sesame functional genomics research (Fig. 2). The phenotypic information is included in the *Material* section, while the genotypic resources are contained in the *Genome related*, *Gene* and *SSR* sections. The BLAST and Genome Browser functions are included in the *Tool* section. The *Download* section provides the genome sequences and genomic variations. The introduction and user guidelines of the database can be found in the *Home* and *Help* sections.

The basic information of the sesame core collections contains 705 sesame accessions that are available on the *Germplasm* page, including the accession name, geographic origin, ecotype, sequencing coverage and group (Fig. 2B). For each information cluster, when clicked, the database provides a drop-down menu with a list of selectable options. It is easy to determine the information for any group of the sesame core collections on this page, which makes it convenient to get the materials for functional genomics research in further studies. The phenotypes of 56 agronomic traits for these sesame accessions are provided on the *Phenotype* page. The minimum, maximum and average values of all traits in the four environments were calculated and are provided on the *Query by trait* page. A fuzzy search function was developed and can be used in the inquiry of phenotypes. Phenotypic value of each trait in each accession can be queried freely on the *Query by accession* page. The plant photos of the accessions can also be viewed on the *Phenotype photo* page.

SesameFG provides three genomes and massive genome variations in the *Genome related* section. Based on the alignment of the three genomes, collinear regions between the sesame landrace and variety genomes can be queried by limiting the genomic coordinates of the “Zhongzhi13” genome on the *Genome comparison* page. The SNPs, Indels and transposons that were identified from the genome comparison of the three sesame genomes are available on the *SNP query*, *Indel query* and *Transposon query* pages, respectively. All of these genome variations can be easily searched by limiting the genomic coordinates of the sesame genome (Fig. 2C). The linkage group location, position, number in the different groups and frequency in different groups of population SNPs are also available. The SNPs in a specific region, around a chosen SNP and around a chosen gene can be queried on the *Population SNP query* page. Moreover, the SNPs between each of the two accessions are provided on the *Query SNP between two accessions* page.

The sesame gene families, gene expression profiles and candidate genes of important agronomic traits are available in the *Gene* section. Detailed information regarding the gene families is provided on the *Gene family* page (Fig. 2D). Thus far, there are 29 gene families with 4,085 members that can be found in the database, including transcription regulators, kinase protein-encoding genes, cytochrome P450 proteins, and lipid metabolism enzymes. Detailed information regarding the gene families can be used in functional analyses of the gene clusters. For example, the identified MADS-box genes are involved in the photo-period regulation of sesame flowering^17^. Expression profiles of sesame genes in the root, stem, leaf, capsule and seeds, which are valuable for gene identification, are all available on the *Gene expression* page. These gene expression results are crucial for gene family analysis and gene function validation. For instance, gene expression profiling of the Hsf gene family had been analyzed based on the data provided in this database^37^. Detailed information of the candidate genes with important agronomic traits can be obtained on the *Candidate genes* page, including the Gene IDs and locations of the genes in sesame genome, the related traits, the peak SNP associated with the related traits, the major allele of the peak SNP, the major allele frequency of the SNP and annotation of the candidate genes. Since only a few genes have been validated by population mapping and molecular experiments in the sesame genome, these candidate genes can be valuable resources and greatly promote sesame functional genomics research.

The SSR loci in the sesame genome, SSR markers design function and polymorphic SSR markers all can be found in the *SSR* section. All 104,836 SSR loci and primer sequences are available on the *SSR Query* page (Fig. 2E). The SSR markers can be queried and designed over a random range or for a chosen gene. The polymorphic SSRs that were detected from previous experimental research are all provided on the *Polymorphic SSRs* page.

SesameFG provides tools to facilitate bench work and further analysis in the *Tool* section, containing Genome Browser and BLAST. We implemented 10 tracks in the Genome Browser, including gene structures, tRNA genes, microRNA genes, transposons and population SNPs. Users can browse detailed information of each feature of each track on the *Genome Browser* page (Fig. 2F). Detailed information of the genome components and population SNPs can be obtained by clicking the corresponding hyperlink of the feature. Take the population SNPs as an example, the SNP ID, position, reference SNP and alternative SNP, major allele and major allele frequencies in landrace and variety, and the number of SNPs in landrace and variety are all shown in the database. A standard NCBI BLAST software package was embedded in SesameFG, providing a similar sequence search function for the users. Not only is the reference sesame genome available, but the genome sequences of the two landrace accessions are provided on the *BLAST* page. The queried nucleic acid or amino acid sequences can be uploaded in a file or pasted in the search box directly. Several BLAST programs are available for different sequence types. The BLAST function aids users in extracting homologous genome components and annotations of query sequences by quick match.

SesameFG also offers users the capability to download and use the sesame genome sequence and variation data in the *Download* section. The genome sequences include commonly used and requested data sets such as Genome FASTA, Generic Feature Format 3 (GFF3) containing the annotated gene models, CDS FASTA, protein FASTA and transposon GFF of the sesame landrace accessions. These genome sequences can be downloaded from the *Genome sequences* page (Fig. 2G). On the *Genome variations* page, the population SNPs that were generated from the re-sequencing of 705 sesame accessions are provided for downloading.

### Application of SesameFG

**Figure 3:**
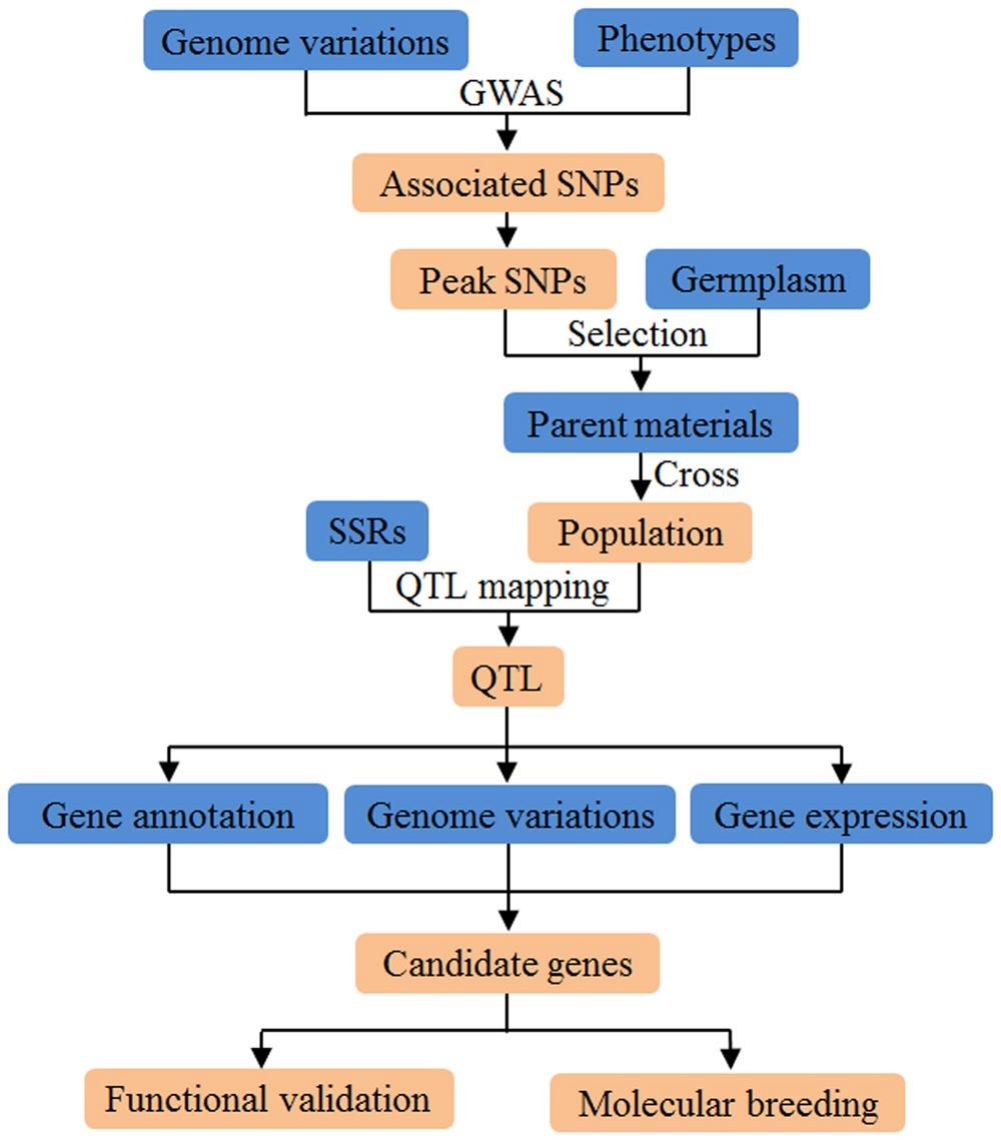
Flowchart of the identification of seed coat color genes from sesame with SesameFG. SesameFG provides user-friendly phenotypic and genotypic information and analysis tools for gene discovery and molecular breeding of important agronomic traits. The blue color represents the information that is available in SesameFG. The orange color indicates the analysis and experimental results.

Seed coat color is one of the most important characters of sesame seeds. There are two major colors of sesame seed: black and white. It has been reported that the *PPO* gene, which encodes polyphenol oxidase and produces black pigments is the key regulatory gene of sesame seed coat color^5^. Here we show that the SesameFG can be easily used in the cloning of *PPO* (Fig. 3).

The sesame coat colors of 705 sesame accessions can be obtained from *Phenotype query*, and SNPs of the sesame accessions can be downloaded from *Genome variations*. GWAS of sesame coat color can be performed using phenotypes and SNPs. Then, the associated SNPs with high degrees of confidence will be gained. Detailed information of the associated SNPs can be queried in the *Population SNP query*. It was revealed that the associated peak SNP at the 11,607,534 bp in linkage group 4, which had a maximal P value (P = 9.33 × 10^−130^), had a C/A mutation; the C allele was related to the black seed coat^2^. Accessions with the black (C) and white (A) alleles can be selected, and F2 populations can be developed by crossing the accessions. SSR markers around the peak SNP (from 100 kb forward to 100 kb behind) can be queried from the *SSR query*. QTL fine mapping of sesame seed coat color can be performed based on SSR markers, and a major QTL containing the peak SNP can be focused into a small region. Then, genes in this QTL can be annotated on the *Literature annotation* page, and the expression profiles of sesame seed genes can be queried on the *Gene expression* page. Since the variety “Zhongzhi13” is a white seed accession and the landrace “Mishuozhima” is a black seed accession, variations of the genes can be found using *BLAST*. Gene annotation, expression and variation analyses showed that only the *PPO* gene was related to the sesame seed coat color. The SSR markers that closely linked to seed coat color can be useful molecular markers for the molecular-assisted breeding of sesame varieties with black seed. Therefore, SesameFG can be effectively used in candidate gene identification and validation of important agronomic traits for sesame. To help new users who want to quickly become acquainted with SesameFG, a step-by-step tutorial of the gene cloning is provided in the *Help* section, with seed coat color is used as an example.

## Discussion

To our knowledge, SesameFG is the only website that provides comprehensive resources related to sesame functional genomics research supported by user-friendly interfaces and an easy-to-used system for the mapping and cloning of important genes. SesameFG was constructed using both phenotypic and genotypic information and based on the first version of the sesame genome^6^. Although three sesame genomics-related databases have been constructed, only the “Zhongzhi13” genome sequence and population SNPs are provided. The genomic-related data available in the databases were lack of integration and functional genomics data, such as QTLs, gene expression profiles, SSRs, and important genes, were not included. The data in SesameFG were collected from these databases and other public databases, literature and research results of the sesame functional genomic research consortium. To improve the usability of the massive amount of data, the sesame genomics and functional genomics-related data were analyzed and integrated in SesameFG. We hypothesized that these data could be conveniently and effectively utilized in discovering the genes of important agronomic traits and developing effective markers for the molecular breeding of sesame.

Compared with cereal crops, such as rice, wheat and maize, functional genomics research of oilseed crops is quite limited. However, the consumption and market demand of vegetable oil has increased greatly in the last decades^44^. With its high oil content (58% in seeds) and small diploid genome (350 Mb), sesame is regarded as an attractive model species for oilseed crops functional genomics research. Previous studies revealed that the major loci underlying oil content in sesame are not always the enzymes in the oil biosynthetic pathway. The genes regulating the non-oil components (mainly protein and dietary fiber) in oilseeds may have important indirect effects on the oil content^2^. This result provides a new strategy in the improvement of the oil content in sesame and other oilseed crops. Therefore, large-scale genetic resources and a database that can be convenient used will provide strong support to the functional genomics research of all oilseed crops.

Although more than 35,000 accessions of sesame have been conserved in the major crop germplasm genebanks of the world and their important agronomic traits have been investigated^30–32^, little phenotypic information of the sesame germplasm is available online. Therefore, it is quite difficult for sesame researchers to find a superior germplasm used for functional genomics research from the existing gene banks. A database containing detailed phenotype information of various germplasms is greatly needed for the sesame researchers. To data, this is the first time that the phenotypes of sesame germplasms have been freely available on a website. The detailed phenotypic information of these materials will be useful for sesame researchers in selecting materials. In addition, all of the germplasms in this database have been genome sequenced and their SNPs are also provided, making the germplasm easily selectable for use in GWAS, evolution, QTL mapping, gene cloning and molecular breeding studies. Of note, this database contains the most comprehensive phenotypic and genomic information for oilseed crop functional genomics research published to date and is expected to have a lasting impact on the genomics research and genetic improvement of oilseed crops.

In SesameFG, three high-quality assembled sesame genomes are available. A genome from a single variety does not adequately represent the diversity contained within a species; several accurate genomic sequences are critical for functional genomics research^45^. The three sequenced accessions, “Zhongzhi13”, “Mishuozhima” and “Baizhima”, can be used as model parents of artificial populations. With the three reference genomes and identified genomic variations, the map-based cloning of genes related to complex traits will be greatly accelerated^5^. Heterosis, which refers to higher yield in F1 hybrids compared to the parents, could reach 30–60% in sesame^46, 47^. However, the genetic mechanism of sesame heterosis remains unknown. The availability of three genomes from three diverse sesame accessions provides the opportunity to explore the biological basis of heterosis in sesame. Genomic variations between each two accessions in the sesame core collections have been identified and are available in the SesameFG. This information can also be used in map-based gene cloning when the sesame core collections are selected to be the parents of some populations. These genomes and genomic variations will be very useful for sesame genetic improvement to help meet human’s increasing consumption of vegetable oil.

In addition to the genomes and genome variations that can provide clues for gene cloning, the genetic resources available in the *Gene* section also provide valuable information for identifications of gene function. Combined with the gene families and gene expression profiles, genes involved in the regulation of some agronomic traits, such as flowering, waterlogging resistance, and drought tolerance, can be discovered^16–18, 37^. The candidate sesame oil-related genes in the database (e.g., *SiNST1*, a gene involved in lignin and cellulose biosynthesis) likely also play important roles in other oilseed species, offering the opportunity to look for genes with common functions in these other species.

Although SSR markers are widely used in high-throughput genotyping and map construction for high-abundance, high-polymorphism and stable co-dominance, -SSR makers for sesame functional genomics research are lacking. Based on the first sequenced and assembled sesame genome, all genomic SSR loci were surveyed and identified in the SesameFG. The polymorphic SSR markers have also been collected and are available. Among these SSR markers, thirty-two SSR markers were selected as the core SSR markers and have been successfully used in the genealogical analysis of sesame elites^9^. The SSR design tool, which is included in the database, will substantially accelerate the QTL and gene fine mapping of sesame. These SSR markers can provide useful resources for genetic linkage map construction, genetic diversity detection, evolution analysis and marker-assisted selection breeding of sesame.

Seed coat color is an important agronomic trait in sesame. Through the utilization of the phenotypic and genotypic resources in the database, the candidate gene *PPO*, which encodes polyphenol oxidase and produces black pigments through a browning reaction, was identified as the key regulation gene of seed coat color in sesame. Several indels were identified in the coding regions of *PPO*, resulting in loss of function of the *PPO* enzyme, which caused the inability of black pigments to accumulate in the seed coat^5^. In fact, besides the *PPO* gene, several other causative genes related to important agronomic traits have been identified with the help of SesameFG. For example, *SiACS* was identified as the causative gene of capsule number per axil^2^. A GWAS revealed that the peak SNP (P = 1.02 × 10^−128^) can explain up to 60% of the phenotypic variation and is located in the *SiACS*. The SNP resulted in a phenylalanine mutated to a serine at the 284th amino acid of the *SiACS* protein. SSR markers have been designed with SesameFG and used in the QTL mapping of the F2 population, which was generated from crossing the “Zhongzhi13” (three-capsule allele) and “Baizhima” (one-capsule allele) varieties. The QTL region was successfully focused into 79 kb. BLAST results showed that the SNP in *SiACS* was the only coding variant around the QTL region between “Zhongzhi13” and “Baizhima”. For ongoing efforts in the gene cloning projects of more complex agronomic traits for functional genomics research in sesame, this database may provide valuable information and may prove to be an effective assistant system.

Molecular breeding is considered to be the best option for crop breeders to improve breeding efficiency^48^. Three steps require the assistance of massive molecular markers: gene or QTL identifications, formulation of the ideal genotype, and efficient molecular breeding^49–51^. The SNPs that were identified in the genome comparison were collected and used as effective markers in the molecular breeding of rice, soybean and peanut^48, 52, 53^. However, because of the lack of an efficient genotyping system, the molecular breeding of sesame has been difficult in recent years. The SSR markers and SNPs that are closely linked or associated with the agronomic traits provided in SesameFG can be easily obtained and used in molecular breeding of sesame. A high-density SNP genotyping array for sesame molecular breeding can also be designed from the genomic SNPs in SesameFG.

We will continue to make efforts to improve and update the database with newly released sesame phenotypic and genotypic information. The reference genome used in SesameFG will be updated according to new versions of the sesame reference genome sequence. The size of the database will continue to expand with the addition of more sesame genome sequences, genome variations, gene expression profiles, gene families and candidate genes. We are also planning to add metabolomics data into SesameFG, making it a more comprehensive database for sesame functional genomics studies. We will also strive to make the database more user-friendly and more efficient following reflection and feedback on the first version of SesameFG. We hope that the accumulation and spread of sesame phenotypic, metabolomic and genetic information in SesameFG will greatly facilitate sesame functional genomics research and accelerate sesame genetic improvement in the future.

## Conclusion

We developed SesameFG, an integrated repository of comprehensive genotype-phenotype information, to promote sesame functional genomics research. The currently available dataset in SesameFG consists of information on materials and phenotype values, genome sequences, genome variations, gene families, gene expression, candidate genes and SSR markers. The data and functions of SesameFG include valuable material and genetic resources for sesame linkage and association mapping, gene cloning, gene functional validation and evolutionary research. SesameFG will facilitate further functional genomics research, molecular breeding and genetic improvements of sesame, and may also provide useful information in the genetic research of other closely-related oilseed species, such as rapeseed.
